# A Kyojo's Delirium: Insights into the Inner Mind of a Young Asian Female Patient

**DOI:** 10.7759/cureus.6376

**Published:** 2019-12-13

**Authors:** Iain Curtis-Shanley

**Affiliations:** 1 Emergency Medicine, Montefiore Hospital, New Rochelle, USA

**Keywords:** delirium, treatment-resistant schizophrenia, delusional disorder, brain tumor, asian, young female

## Abstract

A young Asian female in her early twenties suffered selective mutism, emotional lability, and delusions during a period of personal crisis. The patient experienced brief intermittent periods of clarity during her psychosis of unknown origin, and wrote “Please save me” on a piece of paper during her first meeting with the author of this report. Her confabulations and delusions are analyzed, along with the family and social circumstances that prevented her from receiving adequate treatment.

## Introduction

In the actual practice of medicine, patients sometimes fail to receive adequate care. This can result from human error and/or institutional/system deficiencies. These can have disproportionally severe effects upon this population because the mentally ill often are highly dependent upon their support systems. When the patient comes from a family that also suffers mental illness and maladaptive traits, the only real support that is left are societal and institutional support structures. If these are also defective, the patient will not receive adequate help.

This case was chosen to be presented to a wider professional audience for a variety of reasons. The details of this young patient's struggles encourage sympathy and understanding towards the mentally ill, remind a professional audience of the value of looking for underlying medical causes to mental illness, and demonstrate the necessity of societies creating safety mechanisms for those who lack adequate support networks. This case also draws attention to the prevalent issue of lack of professional standards in the case of lower-tier providers, which can lead to decompensation in emotionally labile or vulnerable patients [1].

## Case presentation

The patient is a young Asian female in her early twenties. Her chief complaint centered around her recall difficulty and amnesia. She had been hospitalized a few weeks prior by her family owing to her emotionally labile behavior. During this time, she was variously diagnosed by different providers as having bipolar disorder, schizoaffective disorder, or schizophrenia. She had been given high-dose anti-psychotics and sedatives, but her symptoms were refractory to treatment. She fluctuated between cheerfulness and weeping, and possessed a variety of transient delusions, such as belief in magical powers and possessing different identities. Her one persistent delusion was pseudocyesis. The patient resembled the “kyojo” of Noh theater, in that, in addition to her apparent madness, she demonstrated a great predilection for singing.

The patient's past medical history included prior hospitalization for mental illness, although hospital records were inadequate, her family were unreliable narrators and generally uninvolved. She had been hospitalized in eighth or ninth grade as a result of an eating disorder being reported to a school guidance counselor, which resulted in the development of distrust towards people and a year of selective mutism, for the duration of which the patient spoke to no one other than her dog, “Chowder”. According to collateral sources, the patient has currently experienced psychological symptoms for the past two-and-a-half years.

The patient's social history is potentially significant. She was raised in a strict social environment and her parents pressured her to excel academically, which led to her starving herself whenever she felt she had failed to meet their expectations. Her domestic environment caused her to develop a negative body image. She relates having experienced emotional abuse from her parents and her brother. Her family seems to have minimized her initial signs of mental disorder, stating that she was simply tired, and subsequently misrepresented reasons for her hospitalizations, such as that on one occasion, according to medical records, her father stated that she had been hospitalized simply to receive an enema. Her family has previously attempted to hinder her from receiving treatment, although this does not seem to be the current case any longer. She denied any usage of drugs or alcohol. However, her medical record does contain mention of crack cocaine usage, and some of her delusional statements contain mention to a rave, and therefore it seems likely that there was some minor drug usage in the past that the patient doesn't remember owing to her amnesia. She previously worked as a secretary in her father's physical therapy practice, which employment she seems to have discontinued owing to her worsening mental illness. She was previously enrolled in college, and her diary seems to suggest she may have had intended to enter medical school and become a psychiatrist (perhaps in response to increasingly severe symptoms of mental illness) until she suffered a psychotic break.

The patient articulates subjective feelings of mental obtundation and drowsiness. These are likely side effects from the high doses of sedatives that they have been placed upon.

Tests demonstrated that she tested positive for highly elevated white blood cell counts in both her urine and blood. She had numerous phosphate crystals in her urine, but tested nitrite negative. Collateral sources alleged that the patient had a pituitary adenoma.

Her appearance was generally well-kept. Her behavior was variable, usually gregarious but her behavior became slowed and less expressive with the onset of periods of depressed mood. Her speech was typically rapid and somewhat slurred, but she attributed this to her cultural and ethnic heritage. Her voice was usually quite cheerful and lively. The patient's mood was variable, sometimes she stated that she felt fine, although at times she expressed a strong dysphoria. The affect of the patient was often somewhat obtunded and somnolent, likely as a result of medication, but generally lively and expressive and always congruent with her mood. Her thought process was logical, but reasoned based upon irrational beliefs. The patient's thought content was bizarre, but upon closer examination her delusions actually seemed to be drawn from environmental cues and ultimately were confabulations that arose at least partially in response to her memory deficits. Her consciousness was clouded, her concentration was normal, and her short-term and medium-term memory were intact, but she suffered from considerable deficits in her long-term memory. The patient was particularly defective in her episodic long-term memory, but showed some deficits in the semantic component of her long-term memory, such as lacking an explanation for basic phenomena such as reflections and other such things. Patient possessed no auditory or visual hallucinations. Her insight was variable, as she at times demonstrated complete clarity into her condition, and at other times demonstrated minimal insight and chiefly a preoccupation with her delusions. Her judgment was good.

The author believes that this patient has an underlying medical cause to her psychiatric disorder, such as a cerebral neoplasm, which could potentially cause treatment-refractory psychiatric symptoms. The patient otherwise seems to be suffering from unspecified schizophrenia, as various elements of other psychiatric disorders, such as bipolar disorder, are lacking in this particular patient for a variety of reasons. A magnetic resonance imaging (MRI), at least of the brain, either to assess for the presence of a primary tumor or metastases, would have been an excellent choice for this patient. Unfortunately, the psychiatric hospital in which she was managed tacitly declined to pursue this option. Symptomatic treatment of this patient has proven unsuccessful, as she has been refractory to all medication, including even sedation.

The first communication the patient had with this author was notable. The patient was weeping and experiencing a period of mutism, and gestured for a pen and a piece of paper. In lucid handwriting, but on random spots, she wrote: “I have had 24 miscarriages today”, “I am late by 20 months”, “I love Dr Bleau” (“-bleau” was a meaningless suffix she attached randomly to proper nouns, not an actual person). And then she wrote: “Please Save Me”.

The patient resembled the “kyojo” of Noh theater, demonstrating a predilection for singing. She was an interesting and unique patient in many ways, and an excellent teaching case in the value of close observation. She was a young female patient in her early twenties, and this suggested schizophrenia, although peak incidence for this demographic often tends to be later in this decade of life. Superficially, the patient was delusional and disorganized in both speech and behavior, and so this also would seem to suggest a subtype of schizophrenia, specifically, “disorganized schizophrenia” or “hebephrenia”. However, on closer examination, many of these assumptions proved problematic.

The patient lacked auditory hallucinations, or hallucinations of any sort. Her delusions were not the fixed sort that often are seen in a delusional disorder. They were highly transient, usually lasting for no more than 24 hours. Their content was also of interest. One of the patient's delusions was that she was a vampire. The author carefully examined the patient's environment and noted that one of the few books available to the psychiatric patients, and in full and continual display, was a book from a popular contemporary series involving vampires. Similarly, the patient on another occasion, despite having a perfectly intact recall for names, phone numbers, and addresses, on one day maintained that her mother was a character from a famous 1990s sitcom. This was unusual in that she previously had remembered her mother's name and contact details. The author observed the television in the common room for the patients and noted that, throughout the day, it was left on a channel that habitually played that television show throughout the day. These and various other examples demonstrated to the author that the patient's delusions were actually derived from her environmental stimuli. This is a significant observation when coupled with her self-acknowledged issues with her memory. From these two factors, the author concluded that what the patient was actually experiencing was not delusions but confabulation. Confabulation can occur in some psychiatric illnesses, such as schizophrenia, but it more commonly arises in the context of some form of brain damage, such as Korsakoff syndrome. This may indicate that there was some underlying damage to the patient's brain. Since there was some mention of minor drug use in the patient's history, and since the patient made reference to a “rave” in several of her delusions, the author considered it was possible that the patient had experienced brain damage as a result of a drug adulterant. Alternately, drug usage might have been a trigger for the onset of schizophrenia in an already predisposed person.

The criteria of disorganized speech, and also of pressured speech in the case of possible bipolar disorder, is another matter that became suspect upon close investigation. Upon first appearance, the patient had rapid speech that was slurred and disorganized. However, when the author questioned the patient on this, she was aware of these characteristics of her speech, and replied that this was just how people of her cultural heritage spoke. Although in appearance her phenotype was entirely Asian, her actual genealogy was more varied. Her father was Taiwanese, while her mother was Filipino, but of Portuguese heritage, and the patient stated that they spoke in a quick and flowing manner. Her quick and indistinct speech was cultural, not pathological.

One of the patient's most interesting delusions was her pseudocyesis. This delusion is not entirely uncommon in psychiatric patients, so it was not explored any further by many who were familiar with the patient's case. However, given the nature of the patient's other delusions, the author was suspect of this as well. Upon questioning various staff members and examining the matter closer, the author was informed by one of her nurses that the patient was indeed currently experiencing her period. This is likely the origin of the patient's pseudocyesis. The patient lacked the ability to interpret and explain everyday phenomena. Therefore, her mind produced other, delusional explanations. In this case, the abdominal symptoms of menstruation may have been the stimuli that produced the belief in pregnancy, and the bloody discharge of menstruation may have produced the belief in frequent miscarriages. Her delusion of pseudocyesis may thus ultimately be a result of a patient with semantic memory deficiencies responding to the internal stimuli of menstruation, rather than a true fixed somatic delusion resulting from psychosis or psychological illness.

Soon after the author's first meeting with the patient, the author looked into the hospital's records of the patient, who had been hospitalized for several weeks. The patient had been suffering from a long-standing UTI, which had evidently been unnoticed, as no treatment had been undertaken. Urinary tract infections, at least in elderly females, are known to cause delirium. Treatment was then undertaken for the UTI. The patient's baseline mood noticeably improved. However, it did not lead to a complete resolution of their condition.

Since records of the patient were very deficient, and the collateral (the immediate family) was considered unreliable, gathering information on the patient was a task that required creative problem-solving. The two best sources of information turned out to be her diary and analyzing the themes that emerged during conversations with her, the former of these two being of quite some interest as it preserved in permanent form the transition between the patient's normal mind and her psychotic state. The patient used to carry around this relic of her former state with quite some attachment, at least until the psychiatric hospital decided to take it away from her and dispose of it into the trash for unstated reasons.

The author became aware of the patient's diary contents because the patient on one occasion came towards the author and threw it towards him, indicating that he should read it. The patient had already on multiple previous occasions presented the author with samples of her writing or drawings. The patient demonstrated an intriguing contrast between her disordered thoughts and her highly lucid handwriting. She previously presented to the author a drawing of a long, flowing dress. Next to it, there was a heart with “15” written in it. And next to that, there was a name. The patient explained that was her daughter. That same drawing of a dress also later appeared in her diary. The author considered the claim unlikely, as the patient would have had to have given birth while still in elementary school in order for it to be true. She said her daughter hadn't seen her for months, and didn't even know she was here. When then later examining the diary, it demonstrated that the patient had originally possessed notable intelligence and insight, and that she seems to have been taking a course in psychology at the time of writing it. The title of the first page of the diary references the first lecture and Cervantes, the writer of the insane Don Quixote. The patient's decision to enroll in psychology courses may represent an adaptive response to prodromal symptoms. The writing of the diary, originally lucid, began to decay in the course of the writing of the journal. There is a sudden break, and the patient at that point mentions someone wanting to dance happily. On the next page, there is a psychotic drawing of a face. After this, a weird abstract drawing, a sort of cube with multiple cubes inside, which are perhaps abstract representations of a person. Then there are two pictures, one perhaps meant to be a man, one meant to be a cat. The way these figures are drawn is vaguely phallic. Towards the end of the diary, there is a list of online colleges. This is likely another adaptation and evidence that the patient is probably conscious of the strain that is occurring. In one of the pages of the diary, a checklist reads: “Life”, “Shut up!”, “Happiness”, and a check is placed next to all of them.

Her diary seems to suggest that there were prodromal symptoms, but that a sudden psychotic break had occurred. Noticeable was her questioning, written in her notes, of what “healthy” was, and how psychiatry was supposed to help anyone. The latter proved to be a tragic foreshadowing.

After analyzing the diary, the author interviewed the patient for an hour. The author avoided cuing the patient, as her confabulation might cause this to contaminate the results of the interview. Instead, the author guided her rambling and analyzed it for themes.

She said she should be walking down the aisle by now. She said that she didn't remember anything anymore, that she just couldn't hold on anymore, and that everything seemed like one single long memory. The author replied to this that even if she got swept away, there were people here to catch her, because everyone was here to help her.

Her themes included many references to her family. Her father was violent. Her grandmother tried to protect her. Her brother hates her very much. Money is a big issue. They are very poor. She only pays in cash, she doesn't trust credit cards (which the author interpreted as a reference to poverty). She seems to indicate that she had to be the one to take care of her father, and that everyone got angry at her when she didn't. Her father was Taiwanese, her mother Portuguese (but born in the Philippines). She has a strong desire to see her family again. But she says that the basement of her house was made into a Psych ward by her parents. She also believes she is dead. The author suspects this is an expression of depersonalization by the patient.

The author noted during the course of his various discussions with the patient that she retained the ability to spell out things lucidly, perform mathematical operations, recall phone numbers, and recall addresses. When confronted by a person's difficulty understanding her, she became exasperated and assumed the behavior of someone having to explain something that is obvious, which indicates that she probably had little insight into the reasons why people found it difficult to understand her. The author has also speculated that, given that the patient is of immigrant descent, the persona/identity of poverty may be derived from her grandmother's memories. It is also possible the poverty is a relative one, related to living in an expensive neighborhood that is beyond a household's economic means to support. She also expressed that she was a defiant child. Various healthcare providers were of the opinion that she regressed at times. Her paradoxically remarkable memory allowed her to mimic the same tone of voice and words she used when she was little, which was startling to some who observed it.

Owing to records being insufficient, it was difficult to establish an exact timeline for the patient. The patient's hair was dyed blonde about 1 or 2 inches away from her scalp. This indicates a possible chronology, because such a procedure is irregular in a psychiatric ward, and likely the patient would require some degree of cognition and awareness in order to have desired and requested her hair to be dyed. Since hair grows at a rate of around half an inch a month [2], these observations would indicate that roughly 2-4 months ago, the patient had been less severely ill, and that perhaps their current level of functioning was not reflective of their baseline.

Having described the patient's case in more detail, several points become salient. This patient was treatment-refractory to medication. The only effect medication had upon the patient was to cause mental obtundation. The high doses of pharmacotherapy the patient was habitually exposed to had unintended negative consequences. The staff would sometimes become so irritated with the patient's loquaciousness that they would inject the patient with sedatives. However, while most patients would become incapacitated by this, this patient would still be capable of ambulation. The author speculates that this might be a result of their metabolism having adjusted to habitually large doses of sedatives, as she was kept on maximum tolerable doses for all her anti-psychotic medications and sedatives. The use of pharmacotherapy was not effective for this patient, and the reflexive usage of it for all cases is a habit that should be modified.

Other methods were much more effective than pharmacotherapy in modifying this patient. The most noticeable effects came from personal interactions and the development of positive or negative relationships. In one regard, it was important to manage carefully the social environment the patient was exposed to, as the patient would sometimes temporarily and subtly assume the complaints of other patients. The patient is highly affected by her environment, and so it is important to create an environment for her that is sympathetic and free of negative stressors. The patient's reintroduction to her domestic environment seems to have proven a trigger for psychological decompensation. The positive changes that the patient experienced were also in response to personal interactions. During the time the author worked upon the patient's case, their baseline mood experienced a significant improvement, incidents of moroseness and mutism almost entirely ceased, and by reading and observing the subtle cues that the patient gave off and using appropriate interventions, the author was eventually able to prevent any such incidents from occurring while they were present. Most of the patient's acute incidents actually stemmed from frustrations the patient experienced while interacting with the staff, who often resorted to aggressive language and actions as what they regarded as the most efficient way to achieve a result, but which usually trigger acute decompensation for the patient. As such, the greatest treatment for the patient was a positive, empathetic therapeutic alliance, and the introduction to and maintenance of a low-stress environment for the patient. Under these conditions, the patient did not achieve remission of their condition, but would behave in a stable manner and maintain a euthymic mood. Medication, in contrast, failed in this particular patient to achieve noticeable results in any positive metric.

With a patient that suffers this level of impairment, two techniques the author found useful. First, owing to the lack of information, both through records and collateral, and the patient's own issues with amnesia and episodic recall, the gathering of information would normally be a considerable difficulty. However, the author observed if one examined those parts of the patient's delusions that persisted for more than twenty-four hours, and analyzed their general themes, most of the information discerned from there proved to be quite accurate, as confirmed by what information was available on her from her records. This is perhaps not surprising since many parts of the patient's mind were still intact and those of a normally functioning person. It is not impossible that some record of these things remained in the patient's mind, even if not able to be directly recalled, and was able to influence them. Similarly, personal symbolism for this patient remained intact and was actually more effective in this treatment-refractory patient than medications were. Words are of medicinal value and have a chemical effect on people. When the author mentioned “Chowder”, her dog, she not only recognized the name, but her face lit up. This symbol can be used for this patient to elicit this effect. Because of the way the author treated her, the patient became happy every time she saw the author. A pattern of behavior formed a psychological symbol that was able to reliably elicit a chemical effect. Her mood dramatically improved. Alternate treatments are important to consider in a patient like this that isn't responding to medication. It may be useful for doctors who treat such patients to seek the symbols that govern these patients and use them as a form of “endogenous medicine” to treat them. For this patient, for example, “Chowder” (the name of her belovèd dog) is one, and perhaps the long, flowing dress from her diary is another. These symbols can be reliably used to evoke a response, and as such should be considered as part of a patient's treatment, particularly in cases thought clarity has been lost and thus conversational therapy would be less likely to be productive.

Considered in entirety, this patient is a victim of institutional oversight. The patient was misdiagnosed with a wide variety of psychiatric conditions that were based upon a superficial examination of the patient's condition. Pharmacotherapy was initiated and continued despite a clear lack of positive effect, the presence of negative side effects, and the clear expression on the part of the patient that this treatment was hurting her. There was little institutional will to seek more complex, medical causes, or to alter a treatment that was clearly not working. There was no family for this patient willing to advocate for her, and her social worker made no energetic attempt to advocate for her. This is particularly regrettable in that at this early point in her life an adequate intervention would have the highest chance of a successful effect. The patient's treatment was negatively affected by the rapid application of stereotypes and the loss of empathy that occurred as a result. Most of her delusions were ultimately references to television programs or movies, some from her past memories, others from her current environment. As an example, she referred to one of the nurses repeatedly as “Tinkerbell” (a character from the story Peter Pan, which was also made into a popular children's movie). This was assumed to be a hallucination by many of the staff who were treating her. However, upon inspection, the author observed that the nurse's shirt had “Tinkerbell” patterns on it. This was not an example of psychosis, as most who were involved in her care immediately assumed, but rather an act of applying a nickname to a person with whom they were familiar, which is not a psychologically abnormal act.

The patient was much more aware than most of the people who were involved with her care assumed. She was still able to notice many subtle social cues and to utilize complex thinking. When she returned a book, the author turned it around with a rolled up paper that he was holding at the time, in order to see the title. This caused her to remark that it wasn't dirty, and she was offended. On the next day, she tested a theory she had developed on the prior day and presented the author with an object and requested that he throw it out, which would require the author to make direct contact with an object she had touched. This demonstrates that long-term recall, complex problem-solving, and social awareness were in some degree preserved in this patient. On another occasion, the patient commented on the author writing down everything she said, and said it was creepy. When the author looked up, she was able to perceive from subtle facial cues the effect this statement had, and she attempted to pretend it was a joke. This again demonstrates a much higher degree of cognitive functioning than the complete insanity that was generally assumed. And the result of that assumption was that she became dehumanized in the eyes of several of the lower-tier providers and thus they began to behave poorly to her and trigger her to decompensate.

What ultimately was the source of the patient's state is likely to remain unknown. The patient, in her more lucid moments, seems to also have been aware of this. “No, I'm never going to get well” was one of the final things the patient said to the author.

## Discussion

When systems allow for deficiencies to exist, whether human or structural, real patients suffer from it. This particular patient was such an example. Even if her condition could not be ameliorated through medication, she could have easily received better care that would help to stabilize her condition. Since her delusions are not fixed, and stem from confabulation, and are drawn from her environmental cues, it seems advisable that she be placed in an environment free from negative/disturbing themes. Instead, to positively influence her mood, her environmental cues should contain familiar/entertaining themes, preferably derived from media with which she was familiar during her adolescent period, which media she seems most to enjoy. The patient, in general, does not demonstrate fixation upon any developmental period. In order to ameliorate the consequences of her confabulation, it would be advisable to fill her environment with cues directly related to her own life and family (assuming she has a positive relation with them, and they don't distress her), such as photos, and perhaps video/audio recordings of family scenes, preferably involving her own identity. It may be that the patient is found to suffer from difficulty fixing/setting certain types of memories more than others (short-term, long-term, emotional, etc.). In which case, the method that she seems to possess the least difficulty with should be preferred. This might help to stabilize her identity. Alternately, modifying her delusions to include aspects of her reality would not likely prove a useful treatment, as all of her false memories are short-term and usually are gone by the time a day has passed. She does, however, seem to be able to remember certain relationship/emotional memories, such as new bonds formed with people, such as a practitioner or staff, and so perhaps this form of learning might serve as another potential anchor point, along with cultural works, that could be modified in order to incorporate reality and a degree of stability back into the psyche of this patient. What will not be likely to improve her condition is to transfer her for permanent keeping in a long-term facility for the sometimes violently psychotically ill, which is where she has been transferred.

There have been many theories published as to the origin of hebephrenia, and these have ranged from adolescent difficulties in interpersonal relationships, to potential underlying metabolic imbalances [3-4]. Currently, mental illness is often managed primarily through medications, but many patients in psychiatric hospitals often prove treatment-resistant to pharmaceutical intervention [5]. However, individualized care in psychiatry is able to significantly improve outcomes in patients [6-7]. What must be further studied and remedied are the institutional and economic problems that in actual practice tend to prevent patients from receiving individualized care.

## Conclusions

Social and economic factors can lead to mentally ill patients not receiving adequate treatment, particularly in cases that require individualized treatment, such as those that are refractory to pharmacotherapy or patients with an underlying medical cause to their disorder. Patients should be housed in conditions that minimize their stressors in order to maximize their possibility for recovery. For this purpose, it is necessary to ensure that all support staff show restraint in their behavior towards patients in order to avoid becoming a source of negative psychological stressors.
